# Measures of Impact

**DOI:** 10.1371/journal.pbio.0030296

**Published:** 2005-07-19

**Authors:** Hemai Parthasarathy

## Abstract

*PLoS Biology* receives a preliminary impact factor.

**im·pact** (i˘m'pa˘kt′) *n.*

*1. The striking of one body against another; collision.*

*2. The force or impetus transmitted by a collision.*

*3. The effect or impression of one thing on another.*

*4. The power of making a strong, immediate impression.*

*—Dictionary.com*

Open-access publishing was headed on a collision course with traditional models of scientific publishing since well before the Public Library of Science launched its first journal. The force of that collision has seen dramatic shifts in the publishing landscape that include increased support from funding agencies for open-access publishing models and institutional archiving, greater availability of free-access articles and options from subscription-based publishers, and the launch of new open-access journals.

*PLoS Biology* was launched in October 2003, less than two years ago, as an open-access home to the very best in biological research. By any measure, the impact of this launch was noticeable. The online publication of our first issue was accompanied by strong and favorable media attention to our articles. The *New York Times* alone covered articles from nine of our first twelve issues. Content from these issues was downloaded, redistributed, and reanalyzed. In 2004, *PLoS Biology* articles were downloaded more than 1 million times. Because the reuse of open-access content is allowed and encouraged, the only restriction (aside from proper citation of the authors) is the creativity of the user. And with the launch of the journal and the attendant excitement about the content, manuscript submissions and presubmission enquiries rose dramatically.

But why did anyone submit great work to a journal that didn't even exist yet, from a publisher with no established reputation? The answer is that it was on the strength of promises made by our in-house editors and academic editorial board to uphold high standards and rigorous peer review, to launch an open-access alternative to the best journals, and to drive a transformation in scholarly publishing. On that promise, more than 250 authors published the 30 research articles that composed our first three issues. And it is on the basis of those first three issues that Thompson ISI has calculated a 2004 preliminary impact factor for *PLoS Biology* of 13.9. [Fig pbio-0030296-g001]

**Figure pbio-0030296-g001:**
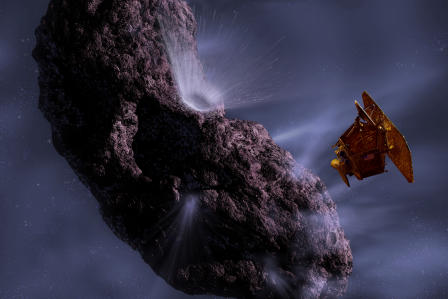
Artist's conception of the Deep Impact spacecraft observing the birth of the new crater on Tempel 1 (Image: NASA/JPL/UMD; art: Pat Rawlings)

Since even before *PLoS Biology* was launched (and plenty of times since then), we've received queries from prospective authors asking about our impact factor. However, because of the way impact factors are calculated, it is not possible to have an impact factor until a specific time has lapsed. Thompson ISI calculates the impact factors that it announces this year by adding up all the citations in 2004 to articles that appeared in a journal in 2002 and 2003, and then dividing the total number of citations by the number of articles published by that journal in 2002 and 2003. For a long-standing journal, therefore, this number reflects the average number of citations over the course of a year to articles published in the two previous years. For *PLoS Biology*, this number therefore refers to citations during 2004 to articles published in only the three months of the journal's lifetime prior to 2004, which is why the initial impact factor can only be considered preliminary.

Of *PLoS Biology'*s article types, Thompson ISI has chosen to define Research Articles, Primers, and Unsolved Mysteries as potentially citeable articles, and, hence, has divided the total number of citations accordingly. As we did not intend the latter two categories to contain articles that would garner citations from the publications monitored by ISI, it does not surprise us that these articles were in fact only cited in scholarly journals 2.4 times on average. Journal editors know that there are various ways to deliberately improve an impact factor, for example, by publishing topical review articles and by weighting content towards more highly cited fields. This begs the question of whether the editors of *PLoS Biology* should play the impact factor game and discontinue some of our educational material in favor of higher citations. On the contrary, our goal is to eventually expand and further develop these components of *PLoS Biology*.

Although our magazine content is an actively evolving section of our young journal, we consider it to be a part of the overall mission of the Public Library of Science to make scientific publishing accessible to more than just the research community. The eventual impact we hope to have on education and policy far outweighs the narrow scope of impact as defined by the impact factor. Our sister journal, *PLoS Medicine*, has outstripped her older sibling in the variety of content designed to educate and spark debate, rather than garner citations. A recently published Policy Forum article, “Nanotechnology and the Developing World” (DOI: 10.1371/journal.pmed.0020097), which identified and ranked the ten applications of nanotechnology most likely to benefit developing countries, was featured in the popular media in nine languages in 22 countries, including reports by BBC and Reuters. While it remains to be seen how an impact factor for *PLoS Medicine* will be calculated, what is more exciting to us is to think about ways to measure impact more broadly.

*PLoS Biology* was launched to give those who believe in the goals of open-access publishing a home for their very best biological research papers, and to show once and for all that open-access publishing is compatible with maintaining standards for the best science. Scientists need to publish in journals that are highly regarded by their peers, and the impact factor is one measure of that judgment. But there are so many more measures of impact. Publishing as a Primer an engaging and personal account by Frans de Waal of the relationship between primatology and sociology (DOI: 10.1371/journal.pbio.0020101) may not have helped our citation numbers, but it will have impacted nonetheless the readers directed to the related paper by an educational supplement in the *New York Times*. In schools and colleges, educators are free to use our content to inspire the next generation to a greater scientific literacy. And in our technological society, scientific literacy is more important than ever.

Comparisons are natural, but the top-tier journals that we aim to challenge were established long before the impact factor was even a twinkle in the eye of ISI's founder, Eugene Garfield. We hope that this number will give those who have wished to support unrestricted dissemination of scientific information, but who have held back for lack of a quantitative measure of the impact of publishing in *PLoS Biology*, one more incentive to submit their best work to this journal. Now is the time to impact the future of scientific publishing for the better.

